# Catheter associated urinary tract infections

**DOI:** 10.1186/2047-2994-3-23

**Published:** 2014-07-25

**Authors:** Lindsay E Nicolle

**Affiliations:** 1 Departments of Internal Medicine and Medical Microbiology, University of Manitoba, Health Sciences Centre, Room GG443 – 820 Sherbrook Street, Winnipeg, MB R3A 1R9, Canada

**Keywords:** Urinary catheter, Bacteriuria, Urinary tract infection, Health care acquired infection, Indwelling urethral catheter

## Abstract

Urinary tract infection attributed to the use of an indwelling urinary catheter is one of the most common infections acquired by patients in health care facilities. As biofilm ultimately develops on all of these devices, the major determinant for development of bacteriuria is duration of catheterization. While the proportion of bacteriuric subjects who develop symptomatic infection is low, the high frequency of use of indwelling urinary catheters means there is a substantial burden attributable to these infections. Catheter-acquired urinary infection is the source for about 20% of episodes of health-care acquired bacteremia in acute care facilities, and over 50% in long term care facilities. The most important interventions to prevent bacteriuria and infection are to limit indwelling catheter use and, when catheter use is necessary, to discontinue the catheter as soon as clinically feasible. Infection control programs in health care facilities must implement and monitor strategies to limit catheter-acquired urinary infection, including surveillance of catheter use, appropriateness of catheter indications, and complications. Ultimately, prevention of these infections will require technical advances in catheter materials which prevent biofilm formation.

## Review

### Introduction

Catheter acquired urinary tract infection is one of the most common health care acquired infections [1,2]; 70–80% of these infections are attributable to use of an indwelling urethral catheter. Recent prevalence surveys report a urinary catheter is the most common indwelling device, with 17.5% of patients in 66 European hospitals having a catheter [1] and 23.6% in 183 US hospitals [2]. In the NHSN 2011 surveillance report, 45–79% of patients in adult critical care units had an indwelling catheter, 17% of those on medical wards, 23% on surgical wards, and 9% on rehabilitation units [3]. Thus, indwelling urethral catheter use is exceedingly common in health care facilities. Prevention of infections attributable to these devices is an important goal of health-care infection prevention programs.

Indwelling urinary catheters are generally considered to be short term if they are *in situ* for less than 30 days and chronic or long term when *in situ* for 30 days or more [4]. Indwelling catheter use in acute care facilities is usually short term, while chronic catheters are most common for residents of long term care facilities. Clinical and microbiologic considerations may vary for short and long term catheters. Urinary catheter acquired infection is usually manifested as asymptomatic bacteriuria (CA-ASB). The term catheter associated urinary tract infection (CA-UTI) is used to refer to individuals with symptomatic infection [4]. In early reports, however, asymptomatic and symptomatic catheter-acquired infection were often not differentiated. This review addresses only indwelling urethral catheters, and will not discuss use of intermittent catheters for men or women, or external catheters for men.

### Burden of illness

#### Asymptomatic bacteriuria

Duration of catheterization is the most important determinant of bacteriuria [4]. The daily risk of acquisition of bacteriuria when an indwelling catheter *in situ* is 3–7%. The rate of acquisition is higher for women and older persons [4]. Bacteriuria is universal once a catheter remains in place for several weeks. Patients with chronic indwelling catheters are assumed to be continuously bacteriuric. From 60–80% of hospitalized patients with an indwelling catheter receive antimicrobials, usually for indications other than urinary tract infection [5]. This intense antimicrobial exposure means antimicrobial resistant organisms are frequently isolated from the urine of catheterized individuals. Statewide surveillance of carbapenemase resistant Enterobacteriaceae (CRE) in Michigan reported 61% of isolates were from urine cultures, and a urinary catheter was present in 48% of these patients [6]. Bacteria colonizing the drainage bags of catheterized patients have been reported to be a source for outbreaks of resistant organisms in acute care facilities [4,7]. In the nursing home setting, the urine of residents with chronic indwelling catheters is the most common site of isolation of resistant gram negative organisms [8,9].

#### Symptomatic urinary tract infection

CA-UTI is the most common adverse event associated with indwelling urinary catheter use (Table 1), although only a small proportion of acute care facility residents with CA-ASB develop symptomatic infection [10]. In the European prevalence survey, 1.3% of patients had urinary infection, representing 17.2% of all healthcare acquired infections, and the third most frequent infection [1]. The presence of any health care acquired infection was independently associated with the number of invasive devices, including indwelling urethral catheters, but the proportion of patients with urinary infections and a catheter was not reported. The recent US point prevalence survey reported urinary tract infection was the fourth most common infection, accounting for 12.9% of health care infections; 67.7% of these patients had a urinary catheter [2]. At one Veteran’s Affairs (VA) hospital, 0.3% of all urinary catheter days involved symptomatic UTI [11]. A comparative British trial evaluating different types of catheters reported rates of CA-UTI of 10.6%-12.6% of catheterized patients, although only 3.2%-5.0% of infections were microbiologically confirmed [12].

**Table 1:** Recent reports describing incidence of symptomatic catheter-acquired urinary infection

**Country (ref)**	**Population**	**CA-UTI rate/1,000 catheter days**
France [13]	ICU	14.8 (1995);
		8.8 (2004)
Germany [14]	ICU	1.39 (before 2000),
		0.83 (2001, 2002),
		0.68 (2003 or later)
15 developing countries [15]	ICU	7.86 (pre-intervention);
		4.95 (post-intervention)
US NHSN [10]	Critical Care	1.2 – 4.1
	Medical	1.5
	Surgical	3.2
	Burn	4.8
	Postpartum	0.5
	Rehabilitation	3.1
Cyprus [16]	ICU	2.0 – 3.0

CA-UTI rates reported in ICU’s in the NHSN hospitals declined by 18.5%-67% among different adult ICU’s between 1990 – 2007 [17] (Table 1). In France, a 66% reduction was reported over a 10-year surveillance period [13]. Some of this decrease is attributable to more intense prevention efforts, but modification of definitions to exclude asymptomatic bacteriuria has also contributed.

In US long term care facilities, 3–10% of residents are managed with chronic indwelling catheters [18]. European surveillance reports describe indwelling catheters being present in 12% of residents in 10 nursing homes in the Netherlands [19], 12.3% in 92 homes in Italy [20], and 10.1% in 40 homes in Germany [21]. The prevalence of chronic indwelling catheters was 7% overall among residents of 78 nursing homes in Sweden, but was 16% for men and only 3% for women [22]. Residents with chronic catheters have an increased risk of symptomatic urinary tract infection. CA-UTI rates of 0–7.3/1,000 catheter days (mean 3.2/1,000) were reported in Idaho long term care facilities [23]. The incidence of fever from a presumed urinary source is 0.7–1.1/100 catheter days, which is three times greater than observed for residents with bacteriuria but without a urinary catheter [24,25].

#### Bacteremia

Less than 3% of subjects with CA-ASB develop bacteremia with the urinary isolate [10] but, given the high frequency of indwelling urinary catheter use, CA-UTI is one of the most common causes of secondary bloodstream infection in acute care facilities. During a 3 year period in Quebec, 21% of health care acquired bloodstream infections were from a urinary source, and 71% of these were device associated. The incidence was 1.4 urinary bloodstream infections/10,000 patient days. All cause 30 day mortality in patients with CA-UTI bacteremia was 15% [26].

CA-UTI is the source of over 50% of episodes of bacteremia in long term care facilities [4,27]. The risk of bacteremia in residents with indwelling catheters in these facilities is 3–36 times that of residents without an indwelling catheter [28].

#### Other morbidity

Additional infectious complications, usually identified in patients with a chronic indwelling catheter, include urinary catheter obstruction, bladder urolithiasis, purulent urethritis, gland abscesses and, for males, prostatitis [24]. Non-infectious complications attributed to an indwelling urinary catheter include nonbacterial urethral inflammation, urethral strictures, mechanical trauma, and mobility impairment [29,30]. Prospective daily catheter surveillance in a VA centre identified genitourinary trauma caused by the indwelling catheter on 1.5% of catheter days [11].

Several studies report an association of CA-UTI with increased mortality and prolonged length of stay in acute care facilities. For critical care unit patients, these associations are likely attributable to confounding by unmeasured variables with little, if any, mortality directly attributable to CA-UTI [31]. Long term care facility residents with chronic indwelling catheters have an increased mortality relative to residents without a catheter, but this observation is also attributable to confounding from variable patient characteristics, rather than directly attributable to urinary infection [32].

### Pathogenesis of infection

#### Biofilm

Biofilm formation along the catheter surface is the most important cause of bacteriuria [33]. Biofilm is a complex organic material consisting of micro-organisms growing in colonies within an extra-cellular mucopolysaccharide substance which they produce. Urine components, including Tamm-Horsfall protein and magnesium and calcium ions, are incorporated into this material. Biofilm formation begins immediately after catheter insertion, when organisms adhere to a conditioning film of host proteins which forms along the catheter surface. Both the interior and exterior catheter surfaces are involved. Bacteria usually originate from the periurethral area or ascend the drainage tubing following colonization of the drainage bag. Only about 5% of episodes of CA-ASB follow introduction of periurethral organisms into the bladder at the time of catheter insertion.

Organisms growing in the biofilm are in an environment where they are relatively protected from antimicrobials and host defenses. A single species is usually identified with the initial episode of bacteriuria following insertion of an indwelling catheter. If the catheter remains *in situ* and a mature biofilm develops, polymicrobial bacteriuria becomes the norm. For individuals with long term indwelling catheters, 3–5 organisms are usually isolated [34,35]. The microbiology of biofilm on an indwelling catheter is dynamic with continuing turnover of organisms in the biofilm while the catheter remains *in situ*[36]. Patients continue to acquire new organisms at a rate of about 3–7%/day.

The determinants of CA-UTI are not well described. However, catheter trauma or catheter obstruction are well recognized precipitating events. Risk factors for bloodstream infection from a urinary source in acute care patients are reported to be neutropenia, renal disease and male sex [37]. Bacteremia is not a significant complication of chronic indwelling catheter replacement [28].

#### Microbiology

The most common infecting organism is *Escherichia coli*[4]. Other Enterobacteriaceae as well as *Enterococci* spp, coagulase negative *Staphylococcus*, *Pseudomonas aeruginosa*, other non-fermenters, and *Candida* spp are also frequently isolated [24]. Antimicrobial-resistant organisms are common. The urine of patients with indwelling catheters is the major site of isolation of resistant gram negative organisms in both acute and long term care facilities, including extended spectrum beta-lactamase (ESBL) producing Enterobacteriaceae [8] and CRE [6]. *E. coli* is usually the most frequent species isolated from bacteremic CA-UTI patients in acute care facilities (Table 2). However, *Enterococcus spp* (28.4%) and *Candida spp* (19.7%) were reported to be most common at one US tertiary care academic centre [38].

**Table 2:** Species isolated from bacteremia attributed to catheter-acquired urinary infection

		**% of isolates**				
**Population (ref)**	US* [38]	**UK **[39]	**Quebec **[26]	**US **[40]**	**Europe **[40]**	**Spain **[41]
*E. coli*		43.4%	47%	69.3%	71.3%	42%
*Klebsiella spp*		7.5%		16.7%	11.2%	15%
*Enterococcus spp*	28.4%	6%	8%			12%
*P. mirabilis*		13.3%		6.4%	5.0%	7%
*P. aeruginosa*		10.8%			4.1%	12%
*Candida spp*	19.7%		2%			3%

*Tertiary care academic centre.

**Report for gram negative isolates only.

*Proteus mirabilis* is an organism of unique importance for patients with chronic indwelling catheters. This species is seldom isolated following initial colonization of the catheterized urinary tract, so it is not common in patients undergoing short term catheterization [42]. The longer a catheter is in place the more likely *P. mirabilis* will be present. This organism is isolated from about 40% of urine samples collected from patients with chronic indwelling catheters [43]. *P. mirabilis* produces more copious biofilm than other bacteria, and these strains also tend to persist for longer periods of time [36].

Bacterial species which produce urease may facilitate the formation of a crystalline biofilm [44,45]. This material is similar to struvite (infection) stones in patients with urolithiasis. Crusts of this material form along the catheter and are the major cause of obstruction of chronic indwelling catheters. About half of patients with chronic indwelling catheters experience catheter blockage at some time, while some patients experience rapid, recurrent obstruction [46,47]. The urease of *P. mirabilis* hydrolyzes urea several times faster than the urease produced by other organisms [48]. This species is isolated from 80% of obstructed catheters [49]. Other urease producing species include *P. aeruginosa*, *Klebsiella pneumoniae*, *Morganella morganii*, other Proteus species, some *Providencia* spp and some strains of *Staphylococcus aureus* and coagulase negative staphylococci. Urease production by many of these species, including *M. morganii*, *K. pneumoniae*, and *P. aeruginosa*, does not generate an alkaline urine, so these strains are seldom associated with appreciable encrustation on catheters [50].

### Diagnosis of CA-UTI

#### Microbiologic diagnosis

Urine specimens for culture should be collected directly from the catheter or tubing, to maintain a closed drainage system. These may be collected either through the catheter collection port or through puncture of the tubing with a needle [4]. CA-ASB is diagnosed when one or more organisms are present at quantitative counts ≥10^5^ cful/ml from an appropriately collected urine specimen in a patient with no symptoms attributable to urinary infection [4]. Lower quantitative counts may be isolated from urine specimens prior to ≥10^5^ cfu/ml being present, but these lower counts likely reflect the presence of organisms in biofilm forming along the catheter, rather than bladder bacteriuria [5]. A mature biofilm has usually formed once the catheter has been *in situ* for longer than 2 weeks. Urine collected through these catheters are contaminated by organisms present in the biofilm. There is a greater number of species and quantity of organisms isolated than these specimens compared with bladder urine collected simultaneously. Thus, it is recommended that the catheter be removed and a new catheter inserted, with specimen collection from the freshly placed catheter, before antimicrobial therapy is initiated for symptomatic infection [4]. Organisms isolated with quantitative counts <10^5^ cfu/ml from the replacement catheter tend not to persist [51].

#### Clinical diagnosis

The diagnosis of symptomatic CA-UTI is often a diagnosis of exclusion [4,24]. Fever without localizing findings is the usual presentation of CA-UTI. Localizing signs or symptoms such as catheter obstruction, acute hematuria, recent trauma, suprapubic pain, or costovertebral angle pain or tenderness are helpful to identify a urinary source of fever, but are present in only a minority of episodes of presumed symptomatic infection. If localizing genitourinary findings are not present, fever in bacteriuric patients should be attributed to urinary infection only when there are no other potential sources. When the same organism is isolated from both the urine and a simultaneous blood culture, a diagnosis of CA-UTI is presumed in the absence of an alternate source for the bacteremia.

#### Pyuria

Bacteriuric patients usually have pyuria, irrespective of symptoms. Patients with an indwelling catheter may also have pyuria without bacteriuria, as the catheter itself may cause bladder inflammation [10]. Other potential non-infectious causes of pyuria include renal disease, such as interstitial nephritis. Thus, the presence of pyuria in urine specimens obtained from a patient with an indwelling urinary catheter does not identify symptomatic infection in a bacteriuric subject, nor is it an indication for antimicrobial therapy [4,28].

### Prevention of catheter acquired urinary tract infections

#### Guidelines

Several evidence-based guidelines provide recommendations for the development and maintenance of prevention programs for CA-UTI [4,7,52-54]. Approaches to prevention include avoidance of catheter use, policies for catheter insertion and maintenance, catheter selection, surveillance of CA-UTI and catheter use, and recommendations for quality indicators.

#### Program implementation

The facility infection prevention and control program should incorporate measures to limit CA-UTI. Improved outcomes following implementation of these programs have been reported [15,55-57]. The program for a given institution should be individualized to be relevant to local experience, population characteristics, and resources. An essential element of any program is leadership at the senior management level [58].

Infrastructure to support an effective program includes development of policies for catheter indications, catheter selection, and catheter insertion and maintenance [4,7,52]. There must be sufficient staffing and staff education, together with access to adequate and appropriate supplies. A means for documentation of urinary catheter use, including indications and dates of insertion and removal, should be established. Where an electronic patient record is used, documentation of catheter use and automatic reminders for removal should be incorporated into this record. The development and implementation of "bundles" for prevention of catheter acquired urinary tract infections has been described. Introduction of a urinary catheter bundle which included education, catheter insertion and management guidelines, and CA-UTI surveillance, in intensive care units in 15 developing countries was followed by a 37% reduction in CA-UTI rate [15]. A state wide initiative in Michigan introduced a CA-UTI bundle with specific practical recommendations addressing implementation under the concepts of "engage and educate", "execute" and "evaluate" [59].

#### Avoidance of catheter use

The single most important intervention to prevent CA-UTI is to avoid use of an indwelling urinary catheter. There are only a limited number of accepted indications for catheter use [46]:

• Monitoring of hourly urine output in acutely ill patients.

• Perioperative use for selected surgical procedures

Urologic surgery

Surgery on contiguous structures of the genitourinary tract

Large volume infusions or diuretics during surgery

Requirement for intraoperative monitoring of urine output

• Management of acute urinary retention and urinary obstruction.

• To facilitate healing of open pressure ulcers or skin grafts in selected patients with urinary incontinence.

• In exceptional circumstances (e.g. end-of-life care), at patient request to improve comfort.

Alternate voiding management strategies such as intermittent catheterization or, for men, external condom catheters, should be used when possible. Institutional policies should also minimize perioperative catheter use by promoting early post-procedure catheter removal and monitoring of bladder volume with ultrasound bladder scanners, where available, to limit catheter reinsertion for potential urinary retention. When a catheter is indicated, it should be removed promptly once it is no longer required. Patients with indwelling catheters should be identified and reviewed on a continuing basis, preferably at daily rounds, and the catheter removed when no longer indicated. Catheters have been reported to frequently remain *in situ* beyond necessary, sometimes because health-care personnel are not aware the catheter is present [7,52]. A systematic review of catheter discontinuation strategies for hospitalized patients reported that the intervention of a "stop order" to facilitate prompt removal of unnecessary catheters reduced the duration of catheter use by 1.06 days, and use of either catheter reminders or stop orders decreased the CA-UTI rate by 53% [60].

#### Selection of urinary catheter

The smallest gauge catheter possible should be used, to minimize urethral trauma [4,52]. Infection risks are similar with latex or silicone catheters, and whether or not there is hydrogel coating of the catheter. Residents with chronic catheters have a decreased frequency of obstruction with silicone catheters, but this observation is attributed to the larger bore size of the catheter, rather than the catheter material. The use of silver alloy coated catheters does not decrease the frequency of CA-UTI [12,61-63]. Nitrofurazone coated catheters have been reported to be associated with a small decrease in CA-UTI [12], but are accompanied by more frequent catheter removal and increased catheter discomfort. Thus, current evidence does not support the routine use of antimicrobial coated catheters [52].

#### Catheter insertion and maintenance

Recommended practices for catheter insertion and maintenance include [4,7,52].These recommendations are primarily based on consensus, but there is strong evidence supporting a decreased rate of acquisition of bacteriuria by maintaining a closed drainage system. There are no benefits with routine daily periurethral cleaning using normal saline, soap, or an antiseptic [52,64], or with the addition of antiseptics to the drainage bag [52].

• Catheter insertion:

Appropriate hand hygiene

Choice of catheter

Aseptic techniques/sterile equipment

Barrier precautions

Antiseptic meatal cleaning

• Catheter maintenance

Appropriate hand hygiene

Secure catheter

Closed drainage system

Obtain urine samples aseptically

Replace system if breaks in asepsis

Avoid irrigation for purpose of prevention of infection

#### Monitoring of infection

The surveillance of catheter use and complications is important to document the facility CA-UTI rate, the effectiveness of interventions, and to allow comparison with benchmark rates [7,52]. Surveillance with benchmarking was reported, by itself, to decrease infection rates in German intensive care units, although the impact for CA-UTI was not as great as observed for ventilator-associated pneumonia or primary blood stream infections [14]. Standardized surveillance definitions for infection should be used [52]. Core data elements which must be collected to support effective surveillance include recording of catheter indication, catheter insertion and removal dates, urine culture results, and monitoring of bacteremia. Relevant quality indicators are CA-UTI incidence, CA-UTI bacteremia incidence, and the proportion of indwelling catheter use meeting accepted indications.

The outcomes of CA-UTI and bacteremic infection are described using a denominator of device days [52]. However, an effective infection prevention program will minimize catheter use, potentially leading to overall higher device day infection rates as fewer low-risk patients will have catheters [65,66]. Thus, an outcome based on total patient days, the standardized infection ratio, should also be reported [7]. Surveillance data should be reviewed by appropriate individuals and committees, and observations reported back to caregivers on patient wards [7,52].

#### Prevention of CA-UTI in long term care facilities

The prevention of CA-UTI in long term care facilities addresses primarily residents with a chronic indwelling catheter [4,24,28]. There should be frequent, systematic review of any resident with a chronic indwelling catheter to determine whether the catheter remains necessary. Bacteriuria in these residents is not avoidable. Interventions should focus on removing the catheter, whenever feasible, minimizing catheter trauma, and early identification of catheter obstruction. Chronic indwelling catheters should not be changed routinely. They should be replaced only if there is obstruction or other malfunction, or prior to initiating antimicrobial therapy when symptomatic urinary infection is treated [52]. Residents with chronic catheters may use a leg bag for drainage to facilitate mobility. Facility policies should address reuse and cleaning or replacement of the leg bags [67]. Antimicrobial therapy for the treatment of bacteriuria in long term care residents with chronic indwelling catheters does not decrease CA-UTI, but there is an increased isolation of resistant organisms with the antimicrobial therapy. Thus, treatment of asymptomatic bacteriuria should be avoided [24].

## Conclusions

CA-UTI is an important device-associated health care acquired infection. The use of an indwelling urethral catheter is associated with an increased frequency of symptomatic urinary tract infection and bacteremia, and additional morbidity from non-infectious complications. Infection control programs must develop, implement, and monitor policies and practices to minimize infections associated with use of these devices. A major focus of these programs should be to limit the use of indwelling urethral catheters, and to remove catheters promptly when no longer required. Ultimately, however, the avoidance of CA-ASB will likely require development of biofilm resistant catheter materials.

## Competing interests

The author declares that she has no competing interests.
